# Effects of postpartum fatigue, parenting stress, and family support on postpartum depression in Chinese first-time mothers: a cross-sectional study

**DOI:** 10.4069/whn.2024.09.02.1

**Published:** 2024-09-30

**Authors:** Feiyan Yi, Sukhee Ahn

**Affiliations:** College of Nursing, Chungnam National University, Seoul, Korea

**Keywords:** Family support, Fatigue, Parenting, Postpartum depression, Stress

## Abstract

**Purpose:**

This study aimed to explore the levels of postpartum fatigue, parenting stress, family support, and postpartum depression (PPD) experienced by first-time Chinese mothers and to investigate their impact on PPD.

**Methods:**

This cross-sectional survey involved 150 primigravida women attending postnatal checkups in Hebi City, Henan Province, China. Demographic data and information on environmental variables (living conditions, family relationships), postpartum fatigue, parenting stress, family support (expected vs. actual level), and PPD were collected.

**Results:**

The average age of the women was 26.25 years (SD, ±3.90), with 78.7% at risk for PPD (score ≥10). Significant correlations were found between PPD and postpartum fatigue (r=.63, *p*<.001), parenting stress (r=.59, *p*<.001), and family support (r=.40, *p*<.001). In model 1, which examined the influence of women’s demographic variables on PPD, significant factors included a poor relationship with parents (β=.24, *p*=.001), a poor relationship with parents-in-law (β=.18, *p*=.029), and a poor relationship with the husband (β=.20, *p*=.013). When the three research variables were incorporated into model 2, the factors contributing to a higher level of PPD included a poor relationship with parents-in-law (β=.14, *p*=.033), increased postpartum fatigue (β=.37, *p*<.001), increased parenting stress (β=.33, *p*<.001), and less family support than expected (β=.12, *p*=.048).

**Conclusion:**

The most critical factors influencing PPD include postpartum fatigue, parenting stress, poor relationships with parents-in-law, and low family support among Chinese primiparas. To mitigate PPD levels, healthcare professionals should screen mothers for depression in outpatient clinics and offer education and counseling to both mothers and their families or companions regarding PPD.

## Introduction

Postpartum women experience significant shifts in their social and psychological roles as they adapt to motherhood. This transition may lead to unstable mood changes, including depression and anxiety, and feelings of physical and mental exhaustion, compounded by stress related to parenting responsibilities. The prevalence of depression among Korean and Chinese mothers is reported to be 24.6% [1] and 14.6% [2], respectively, at 6 weeks postpartum. Postpartum depression (PPD) can begin as early as 4 weeks after delivery and may occur at any point during the first year postpartum.

Studies have shown that women with PPD often face challenges when returning to work, including difficulties with concentration, impaired working memory, and other issues that affect their job performance [3]. In daily life, PPD not only disrupts the harmony between couples [4] but also impacts the healthy development of infants. Over time, children exposed to their mothers’ negative emotions are more prone to develop traits such as introversion or a reluctance to express themselves [5]. Mothers may also experience excessive self-blame and resentment due to their perceived failures, which can hinder normal breastfeeding practices [6].

The prevalence of PPD varies significantly around the world. In the United States, it ranges from 8% to 23.5% [7], while in South Korea, the rates are between 22.4% and 32.8% [1]. In India, the prevalence stands at 22% [8]. In China, the figures fluctuate between 6.7% and 27.3% [9-11]. More than half of all births in China involve first-time mothers, a trend influenced by the longstanding one-child policy. Despite the shift to a “two-child policy” in 2016, 75.5% of births were still to primiparous women, compared to 24.5% to multiparous women [12]. The prevalence of PPD in China during 2017 to 2018 was 42.1% at 6 weeks postpartum, with a higher incidence of 43.4% among first-time mothers compared to 37.9% among those having their second child [13]. Therefore, research on postpartum adaptation, including PPD, among first-time mothers continues to be necessary.

Variables associated with PPD include postpartum fatigue [14,15], parenting stress [16,17], and family support [18,19], according to studies from Korea and China. Postpartum fatigue is a prevalent symptom following childbirth [20]. The physical and mental exhaustion resulting from postpartum recovery and infant care adversely affects a mother’s ability to perform daily activities [21]. The incidence of postpartum fatigue varies internationally; for instance, 67% of mothers in Japan reported experiencing postpartum fatigue one month after delivery [22], while in South Korea, the figure rises to 82% at 3 months postpartum [14]. In China, 90% of mothers reported fatigue at 6 weeks postpartum, with 51.9% experiencing moderate to severe levels of fatigue. Mothers suffering from postpartum fatigue are at an increased risk of developing PPD compared to those without such fatigue [15].

Parenting stress is strongly associated with PPD [23]. New mothers often face significant parenting stress due to the extensive time, physical effort, and energy required to raise their children [16]. Specifically, stress related to an infant’s temperament and a mother’s lack of parenting experience is a significant predictor of PPD [1,17].

Family support acts as a protective factor against depression, while postpartum fatigue and parenting stress can contribute to increased depression levels. Spousal support is essential for new mothers as they navigate the life changes associated with welcoming a new family member. When husbands actively support their wives and engage in childcare, they not only enhance the marital relationship but also foster a bond with their infants, alleviate the significant childcare burden typically shouldered by mothers alone, and help reduce PPD [1,23]. Furthermore, when couples share the responsibilities of puerperal maternal care and newborn care, it leads to stronger relationships and better child attachment [24]. In contrast, mothers who receive less support from their spouses tend to struggle more with postpartum emotion regulation and experience higher rates of PPD [25]. To prevent PPD, it is crucial that family members provide both physical support, such as help with childcare and household tasks, and emotional support, which includes empathizing with the mother’s needs and caring for one another [23].

Few studies have considered maternal environmental characteristics, such as living conditions and relationships with family members, as factors influencing PPD in Chinese women. In China, it is expected that not only husbands but also biological parents and in-laws will support new mothers during the postpartum period. This support includes caring for the mother, managing household duties, assisting with baby care, and providing direct material support [19,26]. However, the relationships between mothers and mothers-in-law, which are established through marriage, often suffer from a lack of understanding due to cultural differences [27]. These differences can lead to disagreements over child-rearing philosophies and methods, increasing psychological stress. If spouses do not manage these relationships effectively, it can deteriorate the couple’s relationship and severely impact family life, potentially leading to PPD [28]. Another study indicated that a mother’s relationship with her biological parents could act as a protective factor against PPD [29]. A Japanese study observed that mothers living with their in-laws had a 26.9% incidence of PPD, whereas those living with their spouses, parents, or other children experienced a significantly lower incidence of 10.3% [30]. Additionally, research on Chinese mothers showed that the prevalence of depression was 29.4% among those living with biological parents, compared to 54.4% among those living with in-laws [11,31].

Low family income, low education level, and the husband’s unemployment were significantly associated with PPD among Chinese mothers [32,33]. Additionally, a husband’s uncooperative attitude, reduced time spent with the husband, and poorer relationships with the husband, parents, and in-laws were linked to increased rates of PPD [29,32]. Regarding obstetric characteristics, factors such as unplanned pregnancy [15,34], poor infant and maternal health [35,36], and maternal expectations concerning the baby’s sex [37] were also found to elevate the risk of PPD.

Therefore, this study aimed to explore the levels of postpartum fatigue, parenting stress, family support, and depression experienced by first-time mothers in China and to determine their effects on PPD, along with maternal environmental characteristics.

### Research objectives

This study explored the effects of postpartum fatigue, parenting stress, and family support on PPD in first-time Chinese mothers. Its specific objectives were as follows:

1) To determine the levels of postpartum fatigue, parenting stress, family support, and PPD

2) To identify differences in PPD according to participants’ characteristics

3) To investigate the relationships among postpartum fatigue, parenting stress, family support, and PPD

4) To determine the impacts of mothers’ characteristics, postpartum fatigue, parenting stress, and family support on PPD

## Methods

Ethics Statement: This study was approved by the Institutional Review Board of Chungnam National University (No. 202204-SB-050-01). Written informed consent was obtained from the research participants.

### Research design

This study used a cross-sectional design to investigate the factors influencing PPD among first-time mothers in China. This study adhered to the STROBE reporting guidelines (https://www.strobe-statement.org/).

#### Sample and sampling

The study participants were first-time mothers who underwent a postpartum examination 6 weeks after delivering their first healthy baby at the Maternal and Child Health Hospital in Hebi City, Henan Province, China. The inclusion criteria for the study included: (1) being 20 years of age or older, (2) being a first-time mother at 6 weeks postpartum, (3) having no pregnancy complications, (4) being part of a married couple raising their child together, and (5) having the ability to understand and respond to the research questions. The exclusion criteria were as follows: (1) hospitalization of the infant, (2) care of the infant by a non-family member outside the home, and (3) maternal use of antidepressant medication before or during pregnancy, or a diagnosis of depression.

To calculate the sample size required for this study, we set the significance level (α) at 0.05, the power (1–β) at 0.8, and the effect size at 0.15, based on a previous study [38]. We used the G * Power 3.1.9.2 program to determine that a minimum sample size of 118 was needed, considering 10 predictive variables: fatigue, parenting pressure, family support, maternal age, economic level, planned pregnancy, children’s health status, and relationships with the spouse, mother-in-law, and birth mother. Taking into account the participation rate during the corona-virus disease 2019 (COVID-19) pandemic and the need for accurate data, we aimed to recruit a total of 153 participants, which exceeded the initial estimate by 130%.

Of the 180 women who expressed interest in participating in the study, 160 met the inclusion criteria during recruitment, while 20 (11.1%) were excluded for not meeting these criteria. A total of 160 mothers provided written informed consent and completed and returned the questionnaires. However, 10 (6.3%) were excluded due to non-response rates, resulting in a final study population of 150.

### Measurements

#### Postpartum depression

This study utilized the Chinese version of the Edinburgh Postnatal Depression Scale (EPDS) [39]. The reliability of this instrument is reported at 0.76, with a content validity of 0.93 [39]. The revised EPDS comprises 10 items, each rated on a 4-point scale (range, 0–3 points; total score, 0–30). For Chinese women, a cutoff value of 9.5 was established [40], where scores from 0 to 9 are considered normal and scores of 10 or higher suggest a risk of depression. Higher scores indicate more severe symptoms of depression. In this study, Cronbach’s α for internal consistency was .86.

#### Parenting stress

This study utilized the Chinese version of the Parenting Stress Inventory Short Form (PSI-SF) [41]. The reliability of this instrument was reported to range from 0.80 to 0.91, with a content validity of 0.90 [42]. The PSI-SF consists of 36 items, each rated on a 5-point scale (1, strongly disagree to 5, strongly agree). Higher total scores (possible range, 36–180) indicate increased parenting stress. Scores were categorized as follows: 85 or below was considered normal, 86–90 as borderline, 91–98 as high, and 99 or above as very high [41]. In this study, Cronbach’s α was .93.

#### Postpartum fatigue

The Chinese version [43] of the postpartum Fatigue Scale [44] was used. This version demonstrated a content validity of 0.96, reliability of 0.81, and remeasurement reliability index of 0.94 [43]. The 10 items are divided into two dimensions: physical fatigue (items 1–6) and mental fatigue (items 7–10), scored on a 4-point scale (1, never to 4, always). Higher summed scores (possible range, 10–40) indicate higher levels of fatigue. Scores of 10 to 14 are considered to indicate mild fatigue, 15 to 20 correspond to moderate fatigue, and 21 to 40 indicate severe fatigue. In this study, Cronbach’s α was .85.

#### Family support

The Chinese version [45] of the Postpartum Social Support Scale [46], was used to test family support. This scale evaluates both the perceived importance of social support and the actual level of support received. The importance of social support is rated on a scale from 1 (least important) to 7 (most important), while the actual receipt of social support is also measured on a 7-point scale (1, no help to 7, a lot of help). Scores on these scales vary from 34 to 238, with higher scores reflecting greater expectations and satisfaction with the social support received.

Other studies have quantified the social support expectancy gap as the difference between expected and actual levels of social support [19,47]. Accordingly, this study measured family support by calculating the difference between the perceived importance of support and the actual support received. A larger difference score indicates a greater gap in family support expectations, signifying less satisfaction with the social support than expected. Cronbach’s α for the importance subscale was .90 and .93 for actual social support in the Chinese version [45]. In this study, Cronbach’s α values were comparable (.94 for importance and .96 for actual support).

#### General, obstetric, and environmental characteristics

The general characteristics included maternal age, occupation, education, and economic status. Obstetric characteristics covered whether the pregnancy was planned, the mode of delivery (vaginal vs. cesarean), obstetric complications, the baby’s sex, feeding practices, maternal and familial expectations regarding the baby’s sex, and the baby’s health status. Environmental characteristics included satisfaction with living conditions and relationships with the husband, in-laws, biological parents, the husband’s level of attention, and the primary postpartum caregiver.

### Data collection

Data collection occurred from October to December 2022. The researcher outlined the study’s purpose and methodology to the outpatient nurses in the obstetrics and gynecology department, seeking their cooperation. Additionally, a recruitment notice was posted on the hospital’s maternity outpatient bulletin board. Interested mothers who approached the researchers were screened for eligibility, and written informed consent was obtained. The questionnaire set, distributed by the researcher, was completed on-site and took approximately 20 minutes. After completing the questionnaire, the mothers returned it to the researcher. As a token of appreciation for their participation, each mother received a baby hat valued at approximately 5 US dollars.

### Data analysis

The collected data were analyzed using the IBM SPSS for Windows ver. 26.0 (IBM Corp., Armonk, NY, USA), with the level of statistical significance set at less than 5%.

1) Frequency analysis and descriptive statistics were applied to evaluate general and obstetric characteristics, postpartum fatigue, parenting stress, family support, and PPD.

2) Differences in PPD based on general and obstetric characteristics were evaluated using the t-test, one-way analysis of variance, and post hoc analysis.

3) The relationships between postpartum fatigue, parenting stress, family support, and PPD were analyzed using correlation analysis.

4) The impact of factors related to PPD on PPD itself was analyzed using multiple regression analysis.

## Results

### Differences in postpartum depression according to mothers’ general characteristics

The majority of the mothers were aged between 20 and 29 years (80.0%), with an average age of 26.25 years. Approximately half of the mothers had attained a college degree or higher (48.7%), 37.3% were employed, and 65.3% were classified as middle class.

Significant differences in the level of PPD were observed based on maternal age and economic status. Mothers in their 20s exhibited a higher mean PPD score (14.58±5.77) than those aged 30 years and older (11.40±4.69 years; t=2.79, *p*=.006). Additionally, PPD scores among mothers in the middle economic class were lower than those among mothers from the low economic class (13.09±5.27 vs. 15.59±6.32; t=3.26, *p*=.041) (Table 1).

### Differences in postpartum depression according to maternal obstetric and environmental characteristics

Half of the mothers reported having planned pregnancies (50.0%). Two-thirds experienced natural (vaginal) deliveries (66.7%). The average gestational age was 39.49 weeks (±1.66), with 8.7% experiencing birth complications. Over half of the mothers breastfed their infants (56.7%). The distribution of the baby’s sex was nearly even, with boys at 51.3% and girls at 48.7%. A high percentage of mothers (94.0%) reported that their expectations regarding the baby’s sex were met, and 90.0% indicated that the family’s expectations were similarly fulfilled. Most mothers described their health as good (93.3%), and 84.0% reported that their baby’s health was good. Regarding environmental characteristics, more than half found their living conditions unsatisfactory (50.7%). Over 75% of the mothers reported having a good relationship with their husbands, whereas 50.7% described their relationship with their in-laws as poor, and 24.0% reported a poor relationship with their biological parents. The level of spouses’ attention to their wives post-childbirth was high (72.7%).

PPD was significantly higher among mothers who had unplanned pregnancies (t=–2.10), did not meet family expectations for the baby’s sex (t=–2.67), had poor infant health (t=–2.52), had poor relationships with their husbands/in-laws/parents (t=–5.55/–5.65/–5.20, respectively), received low spousal attention after delivery (t=–3.09), and lived in unsatisfactory conditions (t=–3.72) (*p*<.05) (Table 2).

### Levels of postpartum depression, postpartum fatigue, parenting stress, and family support

The mean PPD score was 13.94±5.70, with 78.7% of participants falling into the risk group with scores of 10 or higher. The average score for postpartum fatigue was 20.50±5.33, indicating moderate fatigue. Among the participants, 47.3% experienced moderate fatigue, while 41.3% reported severe fatigue. The mean parenting stress score was 89.93±21.11, suggesting border-line stress levels. Of the participants, 53.3% exhibited higher than normal stress levels, 29.3% had a very high level, and 16.0% experienced high levels of parenting stress. The discrepancy between expected and actual family support was quantified at 26.64±39.49, indicating that mothers received less family support than they anticipated (Table 3).

### Relationships among postpartum depression, postpartum fatigue, parenting stress, and family support

PPD showed moderate positive correlations with postpartum fatigue (r=.63, *p*<.001), parenting stress (r=.59, *p*<.001), and family support (r=.40, *p*<.001). There was also a positive correlation between postpartum fatigue and parenting stress (r=.41, *p*<.001), and a weak correlation between postpartum fatigue and family support (r=.29, *p*<.001). Additionally, parenting stress was weakly but positively correlated with family support (r=.26, *p*=.001) (Table 4).

### Factors influencing postpartum depression

In the hierarchical multiple regression analysis, we initially examined the effects of general characteristics, such as maternal age and economic level, as well as obstetric and environmental factors, including planned pregnancy, infant health status, and relationships with biological parents, husbands, and in-laws, which demonstrated differences in PPD (model 1). Subsequently, we assessed the impacts of the primary study variables (model 2). The data satisfied the criteria for tolerance (value >0.1) and variance inflation factor (value <10), indicating no issues with multicollinearity among the independent variables. Additionally, the Durbin-Watson value was 2.06, confirming the absence of autocorrelation.

Model 1 included seven significant maternal characteristic variables and explained 29% of the variance (F=10.01, *p*<.001). Significant predictors of PPD included a poor relationship with biological parents (β=.24, *p*=.001), a poor relationship with the husband (β=.20, *p*=.013), and a poor relationship with in-laws (β=.18, *p*=.029). Model 2, a more robust regression model, accounted for 57% of the variance (F=21.51, *p*<.001). In this model, the impact of relationships with biological parents and husbands was no longer significant, whereas a poor relationship with in-laws continued to be a significant predictor (β=.14, *p*=.033). Additional variables in model 2 showed that postpartum fatigue (β=.37, *p*<.001), parenting stress (β=.33, *p*<.001), and a discrepancy in expected versus actual family support (β=.12, *p*=.048) were significant risk factors for PPD. The findings indicate that higher postpartum fatigue, increased parenting stress, poorer relationships with in-laws, and receiving less family support than expected are associated with an increase in PPD (Table 5).

## Discussion

This study was conducted among Chinese primiparous mothers to identify the factors influencing PPD. The mean PPD score was 13.94, with 78.7% of participants falling into the depression risk group (scores ≥10). This is significantly higher than the previously reported 34.0% in Chinese first-time mothers at 6 weeks postpartum [32]. This increase may be attributed to the challenges of postpartum adjustment for first-time mothers, compounded by the data collection period coinciding with the COVID-19 pandemic in China and globally. In response to the pandemic, the Chinese government enforced home quarantine and strict control over high- and medium-risk areas [48]. Therefore, mothers were unable to meet with their families and lacked adequate family support compared to pre-pandemic times, potentially contributing to the higher PPD levels observed in this study.

The proportion of mothers reporting moderate to severe postpartum fatigue in this study (88.6%) exceeds the 51.9% prevalence of moderate to severe postpartum fatigue among mothers at 6 weeks postpartum in Shanghai, China [15]. This higher rate is likely attributable to a combination of factors including the mothers’ physical recovery needs during the postpartum period, inexperience with newborn care, round-the-clock caregiving, mental stress and exhaustion, and the lack of family support during the pandemic.

The parenting stress score (88.93±21.11), with 45.3% of participants experiencing high levels of stress, is slightly higher than previous studies: 80.24 from Chinese primiparous women at 6 weeks postpartum [42] and 82.29 from Korean mothers at 6 months postpartum [16]. This elevated stress among primiparous women may reflect lower satisfaction and self-efficacy in parenting. The high level of parenting stress could be attributed to insufficient knowledge about and experience with parenting. Additionally, cultural differences in postpartum care practices between South Korea and China should be considered. According to the Korean Ministry of Health and Welfare’s 2021 Postpartum Care Survey, the utilization rate of postpartum caregivers in Korea was 78.1%; however, even in first-tier developed cities in China, this rate was only 7% to 8%, and it was even lower in smaller cities [49]. Moreover, most Chinese families are extended, with the parents of both spouses living together, likely providing a greater degree of family support than is available to Korean mothers, who are predominantly part of nuclear families. Therefore, compared to the postpartum parenting pressure experienced by primiparous women in South Korea, the pressure faced by their counterparts in China is relatively low.

The family support score was 26.64, higher than the 13.29 recorded in 2016 [47], and comparable to the score of 25.30 observed in 2021 [19]. Higher expectations placed by mothers on their families, spouses, and other close relatives due to their lack of parenting experience are associated with a larger discrepancy between these expectations and the low support they perceive. This discrepancy leads to lower maternal satisfaction with the support received. A gap in expectations of social support can result in negative emotions for mothers, such as frustration and helplessness. Therefore, family members should improve communication with mothers, listen patiently to their needs, show tolerance and understanding, and strive to meet their needs for parenting knowledge, skills, and emotional support. This approach will help narrow the expectation gap, empower mothers to care for their babies independently, facilitate their adjustment to the maternal role, and reduce the incidence of PPD [19].

Among mothers’ characteristics, poor relationships with husbands, in-laws, and biological parents were found to be influential, aligning with findings from previous studies [17,32,35]. For Chinese new mothers, maintaining a favorable relationship with family members, particularly in-laws, appears to reduce the risk of maternal PPD. Therefore, it is crucial to acknowledge that indicators reflecting the sociocultural context and environmental characteristics surrounding the mother can also impact PPD.

The factors making the strongest contribution to PPD in first-time mothers were identified as postpartum fatigue, followed by parenting stress and family support, after accounting for environmental factors such as living conditions and relationships with family members. This aligns with previous research indicating that higher levels of maternal depression are associated with increased postpartum fatigue [38], greater parenting stress [16,17], and lower satisfaction with family support [17,26]. Additionally, this study revealed that poor relationships with biological parents, husbands, and in-laws were significant contributors to higher PPD. Specifically, strained relationships with in-laws, increased postpartum fatigue, elevated parenting stress, and dissatisfaction with the level of family support received were significant factors influencing PPD, even after controlling for environmental factors. The variable concerning the relationship with in-laws remained a significant predictor of PPD in Chinese first-time mothers. Therefore, assessments and interventions targeting PPD should consider the significance of in-law relationships in Chinese culture.

During the data collection period for this study, the Chinese government implemented stringent infection control policies, which included enforcing home quarantine and strictly controlling access to high- and medium-risk areas [48]. As a result, mothers were prevented from meeting with their families and could not receive the same level of family support as they would have during a non-coronavirus period. This lack of support may have contributed to higher levels of PPD. Therefore, it may have been challenging for first-time mothers to adapt to motherhood, exacerbated by this unexpected situational factor that potentially worsened PPD. Given that the level of PPD could have been influenced by the situational crisis of COVID-19, caution is advised when interpreting the results of the study.

Another limitation of this study is that it was conducted among healthy, married, first-time mothers residing in urban areas; therefore, caution should be exercised when generalizing the results. Future research should include comparative studies of PPD among urban and rural mothers, as well as follow-up studies to explore PPD levels after mothers return to work.

Implications for nursing practice include the following strategies. Nurses should perform depression screenings during regular checkups for mothers to identify those at risk for PPD. They should also offer education and counseling to help prevent and alleviate depression. To combat maternal postpartum fatigue and parenting stress, nurses should provide education on newborn care and breastfeeding techniques either at discharge or during postpartum visits to strengthen parenting skills. Additionally, nurses should direct mothers to informational support resources available through social media to bolster postpartum support networks. They should encourage mothers to share household and childcare responsibilities with their partners.

## Figures and Tables

**Table 1.** Differences in PPD according to maternal characteristics (N=150)

Variable	Categories	n (%)	PPD score, mean±SD	t/F (*p*), Scheffe
Maternal age (year) mean±SD, 26.25±3.90	20–29	120 (80.0)	14.58±5.77	2.79 (.006)
	≥30	30 (20.0)	11.40±4.69	
	Yes	56 (37.3)	13.96±5.86	
Maternal occupation	No	94 (62.7)	13.92±5.64	0.04 (.968)
	Secondary school	34 (22.7)	15.03±5.95	
Maternal education	High school	43 (28.7)	13.47±4.28	0.83 (.440)
	University and above	73 (48.7)	13.71±6.29	
	High^a^	3 (2.0)	14.67±3.79	
Economic level	Middle^b^	98 (65.3)	13.09±5.27	3.26 (.041) b<c
	Low^c^	49 (32.7)	15.59±6.32	

PPD: Postpartum depression.

**Table 2.** Differences in PPD according to obstetric and environmental characteristics (N=150)

Variable	Categories	n (%)	PPD score, mean±SD	t/F(*p*), Scheffe
Planned pregnancy	Yes	75 (50.0)	12.97±5.74	–2.10 (.037)
	No	75 (50.0)	14.90±5.54	
Mode of delivery	Vaginal birth	100 (66.7)	13.32±5.73	–1.90 (.059)
	Cesarean birth	50 (33.3)	15.18±5.49	
Obstetric complications	Yes	13 (8.7)	16.23±5.33	1.52 (.130)
	No	137 (91.3)	13.72±5.71	
Baby’s sex	Boy	77 (51.3)	14.21±6.20	0.59 (.557)
	Girl	73 (48.7)	13.66±5.15	
Baby’s feeding practice	Breastfeeding	85 (56.7)	13.47±5.08	0.97 (.382)
	Formula feeding	18 (12.0)	15.44±5.16	
	Mixed feeding	47 (31.3)	14.21±6.86	
Maternal expectations of the baby’s sex	Conforms/Does not matter	141 (94.0)	13.82±5.74	–1.18 (.267)
	Does not conform	9 (6.0)	15.89±5.06	
Family expectations for the baby’s sex	Conforms/Does not matter	135 (90.0)	13.53±5.68	–2.67 (.008)
	Does not conform	15 (10.0)	17.60±4.63	
Maternal health status	Good	140 (93.3)	13.74±5.67	–1.65 (.101)
	Poor	10 (6.7)	16.80±5.75	
Baby’s health status	Good	126 (84.0)	13.44±5.58	–2.52 (.013)
	Poor	24 (16.0)	16.58±5.73	
Relationship with husband	Good	114 (76.0)	12.61±5.28	–5.55 (<.001)
	Poor	36 (24.0)	18.14±4.98	
Relationships with mothers-in-law	Good	74 (49.3)	11.51±5.13	–5.65 (<.001)
	Poor	76 (50.7)	16.30±5.25	
Relationship with biological parents	Good	114 (76.0)	12.68±5.18	–5.20 (<.001)
	Poor	36 (24.0)	17.92±5.53	
Spouse’s attention to the mother	Interested	109 (72.7)	13.08±5.64	–3.09 (.002)
	Not interested	41 (27.3)	16.22±5.29	
Satisfaction with living conditions	Satisfied	74 (49.3)	12.26±4.99	–3.72 (<.001)
	Unsatisfied	76 (50.7)	15.58±5.91	
Postpartum caregivers	Husband	61 (40.7)	13.82±6.18	0.07 (.978)
	Mother	19 (12.7)	14.26±5.54	
	Mother-in-law	50 (33.3)	14.10±5.72	
	Postpartum recuperator	20 (13.3)	13.60±4.58	

PPD: Postpartum depression.

**Table 3.** PPD, postpartum fatigue, parenting stress, and family support levels (N=150)

Variable	Categories	Score	n (%)	Range	Mean±SD
PPD	Normal	≤9	32 (21.3)	2 to 29	13.94±5.70
	Risk	≥10	118 (78.7)		
Postpartum fatigue	Mild	11 to 38	17 (11.3)	11.00–38.00	20.50±5.33
	Moderate	15–20	71 (47.3)		
	Severe	21–40	62 (41.3)		
Parenting stress	Normal	≤85	70 (46.7)	40 to 142	88.93±21.11
	Boundaries	86–90	12 (8.0)		
	High	91–98	24 (16.0)		
	Very high	≥99	44 (29.3)		
Family support, expectations				34 to 238	191.23±34.38
Family support, actual				34 to 238	164.59±42.54
Family support, expected vs. actual				–128.00 to 153.00	26.64±39.49

PPD: Postpartum depression.

**Table 4.** Relationships among PPD, postpartum fatigue, parenting stress, and family support (N=150)

Variable	1	2	3
1. PPD	1		
2. Postpartum fatigue	.63^**^	1	
3. Parenting stress	.59^**^	.41^**^	1
4. Family support, expected vs. actual	.40^**^	.29^**^	.26^*^

PPD: Postpartum depression.

* *p*<.01,

** *p*<.001.

**Table 5.** Factors influencing postpartum depression (N=150)

Factor	Model 1			Model 2		
	β	t	*p*	β	t	*p*
Maternal age_young	.11	1.58	.116	.04	0.84	.403
Economic level^†^	.14	1.97	.051	.01	0.02	.981
Planned pregnancy^†^	.01	0.08	.933	.03	0.60	.548
Baby’s health status^†^	.07	1.02	.310	.04	0.80	.426
Relationship with biological parents^†^	.24	3.26	.001	.07	1.16	.248
Relationship with husband^†^	.20	2.52	.013	.04	0.62	.540
Relationships with mothers-in-law^†^	.18	2.21	.029	.14	2.14	.033
Postpartum fatigue				.37	5.90	<.001
Parenting stress				.33	5.33	<.001
Family support (expected minus actual)				.12	2.00	.048
F (*p*)	10.01 (<.001)			21.51 (<.001)		
Adjusted R^2^	.29			.57		

β, standardized regression coefficient.

† The reference values were maternal age (0=30 years and more), economic level (0=high and middle), planned pregnancy (0=yes), baby’s health status (0=good), relationship with biological parents (0=good), relationship with husband (0=good), and relationships with mothers-in-law (0=good).
